# From Balloon to Brain Ballooning: A Case of Obstructive Hydrocephalus in a Child

**DOI:** 10.1055/a-2803-4470

**Published:** 2026-02-16

**Authors:** Shachar Zion Shemesh, Noa Rennert, Paz Kelmer, Zeev Feldman, Lior Ungar

**Affiliations:** 1 Department of Neurosurgery, Sheba Medical Center, Ramat Gan, Israel; 2 Gray School of Medicine, Tel Aviv University, Tel Aviv, Israel; 3 Dina Recanati School of Medicine, Reichman University, Israel

**Keywords:** cerebrospinal fluid, arachnoid cyst, Valsalva maneuver, endoscopic third ventriculostomy, case report

## Abstract

**Background:**

Intraventricular simple cysts, often termed intraventricular arachnoid cysts, are rare benign intracranial lesions in children and are usually asymptomatic. When they become symptomatic, the presentation is typically related to obstructive hydrocephalus or seizures. Clear clinical triggers for abrupt decompensation are not well defined.

**Observations:**

A previously healthy 6.9-year-old boy presented in stupor with acute obstructive hydrocephalus. The only notable antecedent event was repetitive, forceful balloon inflation the night before, followed by early-morning headache and vomiting. Examination showed papilledema, nuchal rigidity, and positive Kernig and Brudzinski signs without focal deficits. CT revealed triventricular hydrocephalus with a normal fourth ventricle. Cerebrospinal fluid (CSF) and blood studies were unrevealing for infection. Worsening hydrocephalus prompted emergent frontal ventriculostomy. MRI confirmed supratentorial obstructive hydrocephalus with trans-ependymal CSF seepage. Endoscopic third ventriculostomy through the ventriculostomy tract exposed a thin, transparent, vascularized cyst bridging the right lateral ventricle and third ventricle near the foramen of Monro. Endoscopic cauterization and fenestration of the cyst wall restored CSF flow, and the child recovered fully without deficit.

**Lessons:**

This case suggests that repetitive, forceful Valsalva-like maneuvers during excessive balloon inflation can acutely raise intracranial pressure and precipitate decompensation in children harboring an unrecognized intraventricular arachnoid cyst, resulting in acute obstructive hydrocephalus.

## Introduction

Intraventricular simple cysts are benign intracranial lesions that are also referred to as intraventricular arachnoid cysts. Under this term falls a spectrum of cystic lesions, including ependymal cysts and large choroid plexus cysts, which share similar imaging and operative characteristics but differ in the histology of the cyst wall. 1 Prior to the widespread use of modern neuroimaging, intraventricular arachnoid cysts were considered extremely rare in children. As imaging has become more accessible, these lesions are identified more frequently, although the majority remain incidental findings and are clinically silent. 1 2 3

When intraventricular arachnoid cysts do become symptomatic, children may present with focal neurological deficits, seizures, headaches, or clinical and radiographic signs of obstructive hydrocephalus. Most cysts follow a benign natural history, but in rare cases cyst rupture or hemorrhage has been described. 4 5 6 A case-control study in pediatric patients demonstrated that larger maximal cyst diameter and a recent history of head trauma were associated with an increased risk of symptomatic cyst rupture or hemorrhage. 7 8 9

The Valsalva maneuver (VM) results from forceful exhalation against a closed glottis and occurs physiologically during activities such as coughing, defecation, or strenuous effort. VM causes a rise in intrathoracic and central venous pressure, which is transmitted to the intracranial compartment, leading to a significant, transient increase in intracranial pressure. 10 11 In a study of clinically stable neurosurgical patients with ventricular drains, VM performed alone produced a marked elevation in intraventricular pressure at the level of the foramen of Monro.

We describe a child with an undiagnosed intraventricular arachnoid cyst who developed acute obstructive hydrocephalus shortly after inflating a large number of balloons for a family celebration. This case highlights a plausible link between repetitive, forceful VM-like activity and sudden decompensation of a previously asymptomatic intraventricular cyst.

## Illustrative Case

### Presentation and Initial Workup

A 6.9-year-old boy with no significant past medical history was brought to the emergency department in a stuporous state. The only remarkable point in the history was that, on the evening before admission, he had inflated a large number of balloons for a family birthday celebration. He went to sleep and awoke the next morning with a severe headache and repeated vomiting, without fever or visual complaints.

On arrival, his Glasgow Coma Scale score was 9; he was hemodynamically stable but somnolent and intermittently restless. Neurological examination revealed nuchal rigidity and papilledema. He was afebrile on presentation, and admission laboratory testing showed no leukocytosis. His pupils were equal and reactive. Kernig and Brudzinski signs were positive, and there were no focal neurological deficits. The patient was initially evaluated in the emergency department by the pediatric team. Although ventricular enlargement was present on the initial CT scan, it was not interpreted at that time as significant obstructive hydrocephalus and was considered possibly secondary to meningeal inflammation. Given the prominent meningeal signs, including nuchal rigidity and positive Kernig and Brudzinski signs, the working diagnosis was acute meningitis. Accordingly, empiric intravenous ceftriaxone therapy was initiated, and lumbar puncture was performed.

The initial CT demonstrated mild enlargement of the lateral and third ventricles with a normal sized fourth ventricle, without evidence of intracranial hemorrhage, mass lesion, or subarachnoid blood. Subtle meningeal enhancement was noted, further supporting the initial suspicion of meningitis.

A repeat head CT performed subsequently demonstrated marked progression of ventricular enlargement consistent with worsening obstructive hydrocephalus, at which point neurosurgical consultation was obtained and an external ventricular drain was placed without complication.

Lumbar puncture revealed crystal-clear cerebrospinal fluid (CSF) with normal opening pressure (20 cm H _2_ O), protein (27 mg/dL), and glucose (67 mg/dL); direct smear/cultures and a viral PCR panel were negative. Blood cultures were negative, CBC/chemistry was within normal limits without leukocytosis, and Brucella serology was negative. Ventricular CSF sampled later showed similarly noninflammatory studies with negative cultures/PCR.

### Progression and Ventricular Drain

Despite the absence of laboratory or CSF evidence of infection, the child demonstrated ongoing clinical deterioration, including depressed level of consciousness, persistent headache, recurrent emesis, and radiographic progression of supratentorial hydrocephalus. Repeat non-contrast CT demonstrated worsening supratentorial hydrocephalus, without a discrete intraventricular mass; a thin obstructing membrane/cyst near the foramen of Monro remained inconspicuous on CT. Given clinical deterioration overnight with worsening hydrocephalus on repeat CT, on the next day, he underwent right frontal ventriculostomy. An external ventricular drain was inserted, with the drainage level initially set at approximately 20 cm above the tragus. During catheter insertion, after the typical “drop” into the ventricle, the surgeon felt the catheter pass through an additional membranous structure, an unusual intraoperative sensation documented in the operative note. Following ventriculostomy, the boy's clinical status improved. A confirmatory CT scan showed the drain in satisfactory position and partial reduction of ventricular size.

**Fig. 1:**
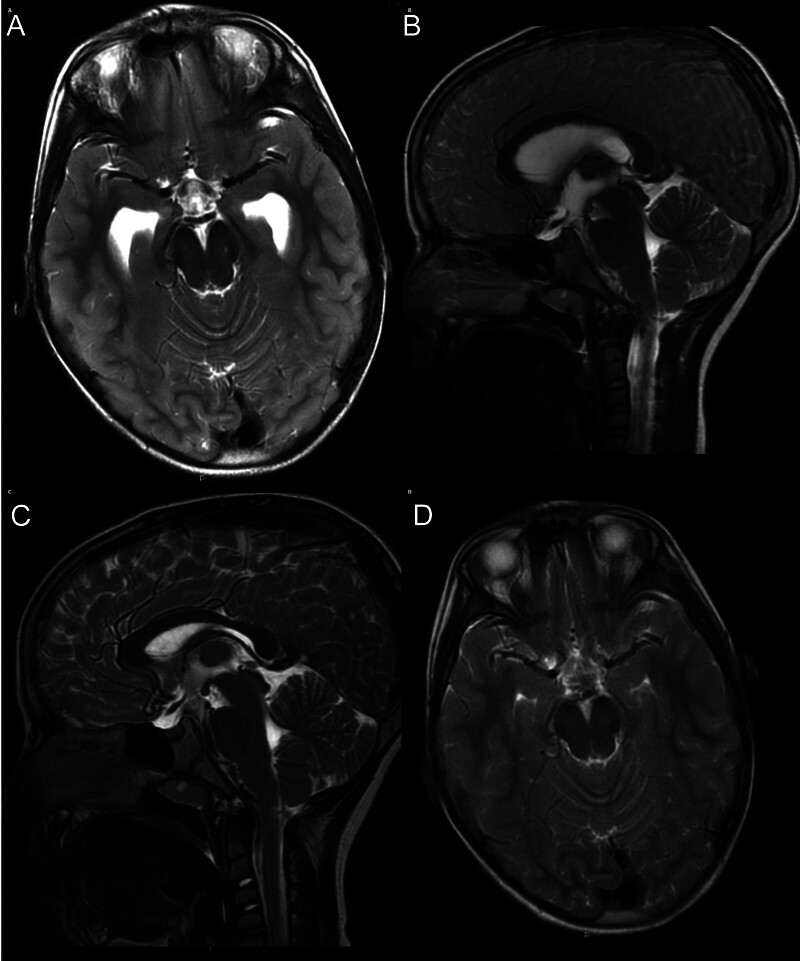
pre-and postoperative MRI demonstrating resolution of obstructive hydrocephalus caused by an intraventricular arachnoid cyst. ( **A** ) Preoperative axial T2-weighted MRI demonstrating marked dilatation of lateral ventricles, consistent with obstructive hydrocephalus. ( **B** ) Preoperative mid-sagittal T2-weighted MRI showing a CSF-intensity cystic lesion in the posterior third ventricle lesion, compressing the tectal plate and aqueduct, with enlargement of the lateral and third ventricles and relative preservation of the fourth ventricle, compatible with triventricular hydrocephalus. ( **C** ) Postoperative mid-sagittal T2-weighted MRI after endoscopic fenestration of the intraventricular arachnoid cyst demonstrating re-establishment of the CSF pathways and interval decompression of the third ventricle. ( **D** ) Corresponding postoperative axial T2-weighted MRI showing marked reduction in ventricular size and resolution of the obstructive hydrocephalus.

MRI of the brain was performed under general anesthesia and was initially interpreted as unremarkable with respect to an obstructing intraventricular lesion. Following lumbar puncture, the patient continued to deteriorate clinically and repeat non-contrast head CT demonstrated further worsening of supratentorial ventricular enlargement. After placement of the external ventricular drain, the patient showed clinical improvement. MRI findings were subsequently re-reviewed after surgical intervention, at which time moderate supratentorial hydrocephalus with ballooning of the temporal horns and transependymal CSF seepage was confirmed. On retrospective review, subtle findings suspicious for a thin intraventricular cyst near the foramen of Monro were identified. The fourth ventricle was normal in size, without tonsillar herniation. T2/FLAIR showed diffuse hyperintensity in the subarachnoid spaces, more conspicuous after gadolinium, raising concern for meningeal inflammation versus elevated CSF protein ( Fig. 1 ).

High-resolution steady-state sequences such as CISS or FIESTA were not included in the initial MRI protocol, as these sequences are not routinely obtained and are primarily used for detailed evaluation of cranial nerves.

Given persistent diagnostic uncertainty, a second lumbar puncture was performed with minimal CSF volume removed given concern for a deleterious pressure gradient in the setting of obstructive hydrocephalus.

### Endoscopic Treatment and Outcome

The patient underwent an endoscopic procedure aimed at creating a stoma in the floor of the third ventricle. The previous frontal incision was reopened, the burr hole was widened, the dura was opened, and the ventricular catheter was cut and removed with sampling of CSF. Using neuronavigation to identify the foramen of Monro, the endoscope was advanced into the third ventricle.

An endoscopic third ventriculostomy was performed by perforating the floor of the third ventricle with a diathermy probe and gently enlarging the stoma with forceps to establish communication with the prepontine cistern.

During withdrawal of the endoscope, the surgical team identified a thin, transparent membranous structure containing fine blood vessels, extending from the right lateral ventricle into the third ventricle. This structure was recognized as an intraventricular arachnoid cyst closely opposed to the ventricular wall near the right foramen of Monro. The cyst wall appeared to have been perforated by the ventricular catheter placed during the prior ventriculostomy, consistent with the unusual tactile sensation noted at the time of catheter insertion. The cyst wall was subsequently coagulated and widely fenestrated endoscopically, resulting in decompression of the lesion and restoration of CSF flow.

Postoperatively, he remained afebrile and neurologically intact, and follow-up lumbar puncture findings were normal. In consultation with infectious disease specialists, he completed ceftriaxone; doxycycline was added empirically for possible zoonotic/vector-borne exposure given prominent meningeal signs and equivocal early leptomeningeal imaging despite repeated noninflammatory CSF. He was discharged home without the need for a permanent CSF shunt. At outpatient follow-up after discharge, the patient remained asymptomatic, neurologically intact, and did not require further CSF diversion.

## Discussion

This case describes acute obstructive hydrocephalus in a previously healthy child with an unrecognized intraventricular arachnoid cyst, in close temporal association with repetitive, forceful balloon inflation. Notably, the obstructing lesion was not appreciated on the initial CT and was only recognized intraoperatively; thin, transparent intraventricular membranes near the foramen of Monro can be radiographically occult and may cause functional obstruction despite the absence of a discrete mass on routine imaging. The clinical sequence and operative findings suggest that repetitive Valsalva-like maneuvers may have precipitated decompensation of a previously asymptomatic intraventricular cyst.

A case-control study of pediatric arachnoid cysts found that larger cyst size and a recent history of head trauma were associated with an increased risk of cyst rupture or hemorrhage. These findings support the concept that mechanical or pressure-related stressors can destabilize otherwise quiescent cysts in susceptible children. 12 13 In the present case, there was no reported head trauma, but there was intense, repetitive VM-like activity in the form of balloon inflation immediately before symptom onset. It is plausible that repeated transient surges in ICP during VM caused cyst distension or altered its position at the foramen of Monro, leading to acute obstruction of CSF flow.

The VM is known to acutely raise ICP. In neurosurgical patients with ventricular drains, VM performed alone has been shown to more than double intraventricular pressure at the level of the foramen of Monro compared with resting values. These transient ICP spikes are usually well tolerated in individuals with normal intracranial compliance, but in the presence of a space-occupying lesion or a pre-existing obstruction in CSF pathways, compensatory mechanisms may be insufficient, resulting in clinical deterioration.

The initial presentation in this child, with papilledema, nuchal rigidity, positive meningeal signs, and altered consciousness, understandably led to a working diagnosis of meningitis. However, repeated normal CSF analyses from both lumbar and ventricular samples, together with the pattern of isolated supratentorial hydrocephalus and a normal fourth ventricle, argued strongly against an infectious etiology. Ultimately, endoscopic visualization confirmed the presence of an intraventricular arachnoid cyst as the mechanical cause of obstruction.

Therapeutically, the staged approach of emergent ventriculostomy for intracranial pressure control followed by definitive endoscopic treatment proved successful. Endoscopic third ventriculostomy combined with cyst fenestration/cauterization allowed restoration of physiological CSF circulation and avoided the need for permanent shunting. This approach is particularly attractive in children, in whom lifelong shunt dependency carries significant risks.

A causal relationship cannot be proven from a single case; the temporal association is hypothesis-generating.

## Conclusion

This case highlights abrupt triventricular hydrocephalus due to an intraventricular obstructing membrane/cyst near the foramen of Monro presenting with meningeal signs and repeated noninflammatory CSF. Although the temporal association with intense balloon inflation is hypothesis-generating and does not prove causality, it reinforces clinical vigilance for occult mechanical obstruction when initial CT is nondiagnostic. Early ventricular CSF diversion for stabilization followed by definitive endoscopic treatment (ETV with lesion fenestration) can restore physiologic CSF pathways and avoid permanent shunt dependence in select pediatric patients.
